# Computed Tomography Findings of Patients Presenting With Headache: 4-Year Retrospective Two-Center Study in Central and Western Regions of Ghana

**DOI:** 10.1155/2024/1833140

**Published:** 2024-09-02

**Authors:** Bashiru Babatunde Jimah, Benjamin Dabo Sarkodie, Asare Kwaku Offei, Ewurama Andam Idun, Dorothea Anim, Edmund Brakohiapa, Benard Ohene Botwe

**Affiliations:** ^1^ Department of Medical Imaging School of Medical Sciences University of Cape Coast, Cape Coast, Ghana; ^2^ Department of Radiology School of Medical and Dental Sciences University of Ghana, Accra, Ghana; ^3^ Department of Surgery Korle Bu Teaching Hospital, Accra, Ghana; ^4^ Department of Radiology Korle Bu Teaching Hospital, Accra, Ghana; ^5^ Department of Midwifery and Radiography School of Health & Psychological Sciences University of London Northampton Square, London, EC1V 0HB, UK

**Keywords:** computed tomography, evaluation, head CT, headache, multicenter analysis, parenchymal lesions

## Abstract

**Objectives:** The radiographic assessment of the head is a crucial part of headache care. A computed tomography (CT) scan enables a more detailed analysis of the condition and more focused care. This study examined head CT scans to determine what kinds of anomalies were present in patients with headaches as their primary complaint.

**Methods:** We evaluated 4 years' worth of CT scan data from head exams conducted at two diagnostic facilities in Ghana's western and central regions. We examined data on 477 patients with a headache as their primary complaint between January 2017 and December 2020. We employed chi-square and Fisher's exact tests (where applicable) to compare head CT diagnoses between age groups, gender, headache subtypes, and brain lesion subgroups.

**Results:** There were 53.5% (*n* = 255) females and 46.5% (*n* = 222) males in the study. The average age of patients was 38.67 ± 17.23 years, with an annual rate of abnormal CT diagnoses ranging from 35.9% in 2017 to 45.4% in 2022. Abnormal head CT diagnoses are strongly correlated with age groups and patient gender (*p* = 0.011 and *p* = 0.009, respectively). Of the 202 patients, 15.3% and 24.3% were classified as intracranial lesions and extracranial lesions, respectively. Maxillary sinusitis affected nearly 60% of the patients, while tumors and hemorrhages affected 25.2% and 11.9%, respectively.

**Conclusions:** A CT scan of the head is essential to detect abnormalities in nearly 50% of patients suffering from various degrees of headache. Sinusitis, brain tumors, and hemorrhage were common lesions detected. It is crucial to create local standard operating procedures to promote better utilization of this type of imaging service, particularly among patients who have been diagnosed with headaches.

## 1. Introduction

A headache is described as a diffuse pain that is not limited to the area of a nerve's distribution [1]. Recurrent headaches are the most frequently reported complaints of the nervous system in routine and emergency medicine [2, 3], which are largely caused by migraines and tension-type headaches [2–4]. However, the secondary causes of headaches are uncommon, the most common of which is medication-overuse headache [3, 4]. Apart from these known causes, head injuries, vascular illnesses, etc. are potential causes. The ophthalmoplegic migraine type has a distinctive clinical presentation, and imaging might not be useful, whereas in others, such as neoplasms, imaging allows for a timely diagnosis and the possibility of treatment [5].

In spite of regional variances, headaches are a global event that affects people of all ages, races, income levels, and locations [2].

About 50% of adults are thought to have a current headache condition, defined as having experienced symptoms at least once in the previous year [2]. Around 50%–70% of adults in the world between the ages of 18 and 65 reported having a headache in the previous year, and at least 30% of those people reported having a migraine [2].

Recurrent headaches are a sign of headache disorders, which also cause social and economic costs as well as personal and societal responsibilities from suffering from pain and impairment [2, 5]. It has been contended that most of the patients suffering from primary headaches can be managed with primary care, with no need for neuroimaging in most cases. It has received inadequate attention worldwide [2]. Only a small percentage of people with headache disorders are correctly diagnosed by a medical professional worldwide [2].

Imaging provides a clinical diagnosis for many headaches [6]. Imaging modality such as computed tomography (CT) remains a primary neuroimaging modality for assessing the various causes of headaches [3, 4, 6]. In developing nations, CT scanning is being used more often for clinical practice and research, with improved features [7]. Many radiologists in Africa preferred CT over other modern but expensive modalities for head trauma investigations due to its low cost and ease of use, among other factors that are advantageous in a resource-limited setting [5, 8].

There are documented reasons to use imaging for headaches, such as an abnormal neurological exam, signs of a systemic illness, headaches getting worse or happening more often, new headaches in people over 50, sudden headaches, new headaches in people with cancer or a weak immune system, and headaches after a head injury [6]. However, studies showed more than 50% of headache cases examined on a CT scan were normal [3, 9, 10]. In southern Ghana, Jimah et al. reported 57.65% of normal CT findings in patients [3], while Ukamaka and Adaorah found 50.8% in Nigeria [10]. Lemmens, van der Linden, and Jellema also reported normal CT in 87% of patients presenting to the emergency department in the Netherlands [9].

Individuals with varying degrees of headache require a radiographic assessment of the head [11–14]. CT allows for a comprehensive diagnosis and prompt, specific care [8]. This study reviewed head CT examinations performed between 2017 and 2020 with the aim of evaluating CT findings among patients presenting with headaches as the principal complaint. Specifically, this study determined the annual distribution and demographic disparities of head CT findings among patients with headaches. We also assessed the association between contrast medium usage and head CT findings and identified the classes of head CT abnormalities among patients with headaches.

## 2. Methods

### 2.1. Study Design and Setting

Between January 2017 and December 2020, the records of 3618 patients who underwent head CT examinations at two diagnostic facilities were reviewed. The study sites were the imaging departments of Cape Coast Teaching Hospital (CCTH) in the central regions and Efiakwanta Regional Hospital in the western regions of Ghana. We kept records of all patients who presented with headaches as their primary complaint at facilities and received their reports from RAAJ Diagnostics. The study excluded patients who had other indications as well as patients with incomplete or missing data.

### 2.2. Data Collection

Records of head CT examinations were reviewed independently by two experienced radiologists, each with more than 8 years of experience in medical imaging. We identified 477 patients' records with headaches as the principal indication for the CT scan. These formed the sample for further review and analysis. Initially, we grouped the sample as either normal or abnormal. Patients with abnormal head CT findings were subgrouped: patients with brain parenchymal lesions (BPLs) only, patients with nonbrain parenchymal lesions (NBPLs) only, and patients who were diagnosed with both lesions (BLs).

### 2.3. Data Management and Statistical Analysis

The data was directly entered into a Microsoft Excel template. Variables captured were the date of request, age, gender, headache subtypes, and type of brain lesions. The data was managed and analyzed using the Statistical Package for Social Sciences (SPSS) software Version 22. We expressed the data descriptively using frequencies and percentages. The trend of head CT examinations for patients with headaches was presented using a linear graph. The proportion of patients with brain lesions or pathologies was compared in terms of age, gender, and headache groups using Fisher's exact and chi-square tests (where applicable) with a *p* value less than 0.05.

### 2.4. Ethical Consideration

The study protocol was reviewed and approved by the CCTH Ethical Review Committee (ERC) (Ref: CCTHERC/EC/2022/059). We maintained data confidentiality and security by replacing patient names with unique ID numbers and using a laptop with a password-protected screen.

## 3. Results

### 3.1. Trend of CT Diagnosis

**Figure 1:**
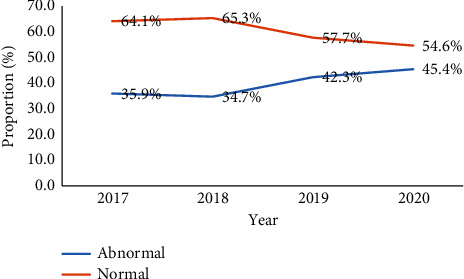
Trend of head CT diagnosis of patients presenting with headache, 2017–2020.

There were 53.5% (*n* = 255) females and 46.5% males (*n* = 222) in the study, with an overall average age of 38.67 ± 17.23 years. There were 272 (57.7%) normal CT findings and 202 (42.35%) aberrant CT findings, with an annual rate of 35.9% (2017) to 45.4% (2020) (Figure 1).

### 3.2. Patients' Characteristics

**Figure 2:**
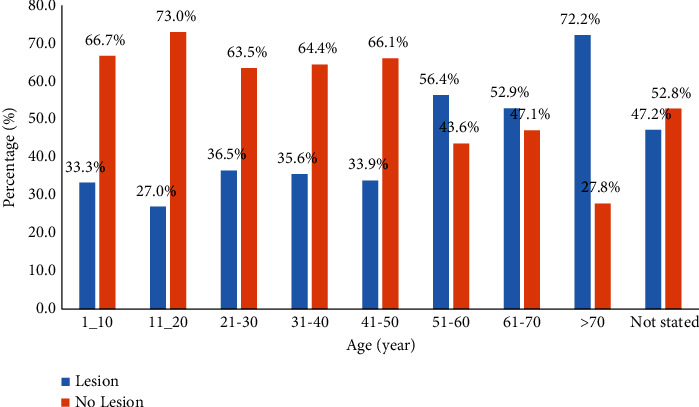
Pattern of CT diagnosis by age patient presenting with headache.

The mean age of patients with brain lesions was 42.99 (±19.01) years older than patients without brain lesions, 35.80 (±15.34) years old. The rate of brain lesions increases with age: 51–60 years (56.4%), 61–70 years (52.9%), and 70 years and above (72.2%) (*p* = 0.011) (Figure 2).

**Figure 3:**
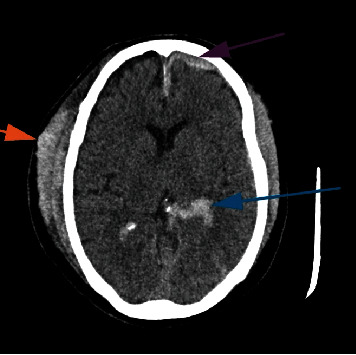
Axial CT scan image showing traumatic hemorrhage (blue arrow, intraventricular; purple arrow, subdural hematoma; orange arrow, scalp hematoma).

**Figure 4:**
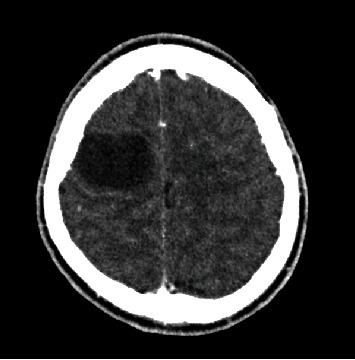
Axial postcontrast CT scan image showing rim enhancing hypoattenuating lesion in the right frontal lobe suggestive of abscess.

Patient characteristics and CT diagnosis are shown in Table 1. The proportion of patients with brain lesions was higher in males (48.6%, 108/222) than in females (36.9%, 94/255) (*p* = 0.009). Patients presenting with headaches from traumatic (60%, 9/15) had a higher rate of brain lesions, followed by those with acute causes (50%, 15/30) (Table 1 and Figures 3 and 4).

### 3.3. Subgroup of Brain Lesions Under CT Scan

**Figure 5:**
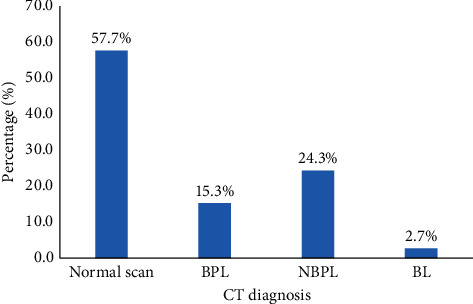
Distribution of patients with headache by a subgroup of brain lesions under CT scan, 2017–2020. BPLs, brain parenchymal lesions; NBPLs, nonbrain parenchymal lesions; BLs, both lesions.

A significant proportion, 42.35% (*n* = 202/477), of head CT examinations were abnormal. Of 202 patients with brain lesions, 15.3%, 24.3%, and 2.7% were classified as BPLs only, NBPLs only, and BLs, respectively (Figure 5).

Nearly 60% of the patients had maxillary sinusitis, whereas 25.2% and 11.9% of the patients had tumors and hemorrhages, respectively. When compared to maxillary sinusitis (90.5%) in NBPL, brain tumor (37%), hemorrhage (28.8%), hydrocephalus (17.8%), and brain infarct were the most prevalent CT in BPL. With the exception of bone-related pathology (*p* = 0.733) and other CT findings (*p* = 0.474), there was a statistical difference between CT abnormal categories (Table 2).

## 4. Discussion

Headache disorders continue to be one of the most common neurological disorders. More than half of the world's population experiences various degrees of headache disorder [2]. A radiographic head assessment is a crucial component of treating individuals with different types of headaches [11–14]. A CT scan facilitates comprehensive diagnosis and permits timely and targeted intervention [8]. This study reviewed head CT scan examinations performed between 2017 and 2020 with the aim of evaluating CT abnormalities diagnosed among patients presenting with headaches as the principal complaint.

Consistent with previous studies [1, 3, 5, 6], a significant proportion of patients in this current study were in the fourth, fifth, and third decades of life. These age brackets are the most productive and engaged in our society, and they are more likely to be exposed to dangers from the workplace and from society as a whole [8]. It was observed that the proportion of males was relatively lower than that of females. The demographic characteristics of patients in the current study are comparable to other data in Ghana [15]. This study revealed that an abnormal head CT scan diagnosis was significantly linked with the age and gender of the patient, contrary to Rai et al. [4], who revealed no significant correlation between demographic characteristics and head CT scan findings in central India.

In this study, 477 head CT scans were performed during the period under investigation. There was a somewhat consistent rise in the number of head CT scans performed among patients with headaches. Likewise, the overall positivity rate was high (42.35%), which increased yearly from 2017 (35.9%) to 2020 (45.4%). The reason behind the extensive use of neuroimaging in headache patients may be the physician's fear of misdiagnosing harmful pathology and the availability of the procedure. Another possibility is that patients want head CT scans, and doctors are more likely to provide them in order to strengthen patient–physician relationships or avoid malpractice claims [9]. However, it must be noted that CT is associated with high radiation, and therefore, it is not advisable to request the procedure without adequate justification.

Generally, NBPL (extracranial lesions) was commonly seen among patients with headaches as compared to BPL (intracranial lesions) [3, 4, 9]. Consistent with this assertion, a previous study by Jimah et al. revealed higher occurrences of NBPL (57.4%) than BPL (36.1%) in Ghana. The findings of the current study show that nearly one-third (24.3%) of the patients presented with extracranial lesions as compared to 15.3% with intracranial lesions. Although the rates were lower, Rai et al. [4] found a comparable pattern of NBPL (19%) and BPL (6%) among patients in central India. The incidence of BPL of 15.3% is comparable to 13% of intracranial pathologies observed in cranial CT scans by Lemmens, van der Linden, and Jellema [9] in the Netherlands. On the contrary, a study by Ukamaka and Adaorah [10] indicated a higher incidence of intracranial lesions (58.1%) than extracranial lesions among patients with chronic headaches in Nigeria. The disparity in findings could be attributed to the types of headache conditions presented by the patients. Also, the influence of differences in geo-sociodemographic features of the study context (in terms of participants, access to facilities and experts, and accuracy of imaging tools) is relevant.

A large proportion of all patients (60%) had maxillary sinusitis, which is consistent with previous researchers linking headache to a separate sinusitis [16–18]. Interestingly, more than 90% of NBPL patients (in Group B) had maxillary sinusitis. Aydemir et al. [19] found a significant link between headache and patients' average maxillary, frontal, and sphenoid sinus volumes, as well as their overall sinus volumes. Although facial pain, facial congestion, nasal obstruction, nasal discharge, hyposmia, or fever (in acute) have been major factors in the diagnosis of sinusitis in adults, the contribution of headaches cannot be overemphasized [16–18].

The incidence of brain tumors or neoplasms is a clinical and public health concern [8, 9]. It is one of the major causes of secondary headache syndromes [2, 9]. In this study, 25.2% of patients with headaches were diagnosed with brain neoplasms, cysts, and metastases. The current prevalence is much higher compared to the 1.8% reported by Lemmens, van der Linden, and Jellema [9] in their study from the Netherlands. The observed disparities in the incidence of brain neoplasm could be attributed to the different tools and protocols used in head CT examinations between the two countries. Also, adequate clinical histories of patients were taken in the Lemmens, van der Linden, and Jellema [9] study, which might inform their interpretations.

### 4.1. The Study Has Limitations

We were unable to group the headache into the globally recognized subtypes due to the clinicians' failure to provide sufficient patient history on the request form. We conducted this study using data from two specialist imaging facilities in southern Ghana, which may limit the applicability of the findings to other facilities in Ghana.

## 5. Conclusion

A head CT scan of patients with headaches at the two centers is a common practice to ascertain the exact causes of headache disorders. Abnormalities were detected in nearly half of head CT scans, which increased over the period of the study. Brain neoplasm, cyst, and metastasis (BPL) and sinusitis (NBPL) were the most common lesions from head CT scans. There was a statistical correlation between head CT diagnosis and demographic factors.

## Figures and Tables

**Table 1:** Characteristics and CT diagnosis of patients presenting with headache.

**Variables**	**Level**	**All patients**	**Presence of lesion**		**X** ^2^ **p** ** value**
		**N** = 477** (%)**	**Yes, ** **n** = 202** (%)**	**No, ** **N** = 275** (%)**	
Sex	Male	222 (46.5)	108 (48.6)	114 (51.4)	0.009^∗^
	Female	255 (53.5)	94 (36.9)	161 (63.1)	

Levels of headache	Acute	30 (6.3)	15 (50.0)	15 (50.0)	0.393
	Chronic	34 (7.1)	15 (44.1)	19 (55.9)	
	Traumatic	15 (3.1)	9 (60.0)	6 (40.0)	
	Not specified	398 (83.4)	163 (41.0)	235 (59.0)	

Abbreviation: *X*^2^: chi-square text.

^*^Statistically significant.

**Table 2:** Subgroups of brain lesions among 202 patients with headache, 2017–2020.

**Diagnosis**	**No. with lesions**	**Proportion (%)**	**BPL, ** **n** ** (%)**	**NBPL, ** **n** ** (%)**	**BL, ** **n** ** (%)**	**Exact ** **p** ** value**
Sinusitis	120	59.4	6 (8.2)	105 (90.5)	9 (69.2)	0.001^#^
Tumor	51	25.2	27 (37.0)	19 (16.4)	5 (38.5)	0.003^#^
Hemorrhage	24	11.9	21 (28.8)	0 (0.0)	3 (23.1)	0.001
Hydrocephalus	15	7.4	13 (17.8)	0 (0.0)	2 (15.4)	0.001
Infarct	14	6.9	12 (16.4)	0 (0.0)	2 (15.4)	0.001
Ischaemic small vessel	8	4.0	6 (8.2)	0 (0.0)	2 (15.4)	0.002
Brain atrophy	8	4.0	5 (6.8)	0 (0.0)	3 (23.1)	0.001
Meningitis	5	2.5	4 (5.5)	0 (0.0)	1 (7.7)	0.028
Bone involvement	4	2.0	1 (1.4)	3 (2.6)	0 (0.0)	0.733
Calcifications	4	2.0	3 (4.1)	0 (0.0)	1 (7.7)	0.044
Others	8	4.0	4 (5.5)	3 (2.6)	1 (7.7)	0.474
Total	202	100.0	73	116	13	

*Note:* Other CT findings include mastoiditis (3), thrombosis (1), diffuse deep white matter (1), enlarged tonsils (1), and herniation (1).

Abbreviations: BLs, both lesions; BPLs, brain parenchymal lesions; NBPLs, nonbrain parenchymal lesions.

^#^Chi-square test of association.

## Data Availability

The data used to support the findings of this study are included within the article.
